# Correction: Impact of the COVID-19 pandemic and typhoid conjugate vaccine introduction on typhoid fever in Nepal

**DOI:** 10.1371/journal.pntd.0014208

**Published:** 2026-04-14

**Authors:** Dipesh Tamrakar, Shiva Ram Naga, Esther Jung, Basudha Shrestha, Pratibha Bista Roka, Rabin Pokharel, Sabin Bikram Shahi, Aarjya Tara Bajracharya, Surendra K. Mahadup, Nishan Katuwal, Kate Doyle, Jessica C. Seidman, Alice S. Carter, Stephen P. Luby, Isaac I. Bogoch, Kristen Aiemjoy, Denise O. Garrett, Rajeev Shrestha, Jason R. Andrews

An additional affiliation is missing for the first author. Dipesh Tamrakar is also affiliated with the Department of Clinical Tropical Medicine, Mahidol University Faculty of Tropical Medicine, Bangkok, Thailand.
