# Supplementary material for: Impact of the COVID-19 pandemic and typhoid conjugate vaccine introduction on typhoid fever in Nepal
Source: PLoS Negl Trop Dis. 2026 Jan 21;20(1):e0013242. doi: 10.1371/journal.pntd.0013242 (PMC12851488; doi:10.1371/journal.pntd.0013242)
Supplement: S1 Table — (DOCX) [file pntd.0013242.s001.docx]

**APPENDIX**

**S1 Table:** Multivariate logistic regression analysis of blood culture positivity for other pathogenic bacteria* by study period and age group (Nepal).

| **Denominator: All blood cultures** | | | |
| --- | --- | --- | --- |
|  |  | **aOR** | **95% CI** |
| 15m-15y | Pandemic v. Pre-pandemic | 0.89 | 0.49-1.53 |
|  | Post-TCV v. Pandemic | 0.39 | 0.18-0.81 |
| ≥16y | Pandemic v. Pre-pandemic | 1.20 | 1.02-1.69 |
|  | Post-TCV v. Pandemic | 0.98 | 0.84-1.16 |

*Other pathogenic bacteria were those determined to be probable pathogens and included *Acinetobacter spp., E. coli, S.* Paratyphi A, *Klebsiella spp., Staphylococcus aureus,* and other organisms.

Abbreviations: aOR, Adjusted Odds Ratio (adjusted for month and study site), CI, Confidence Interval, Pre-pandemic: January 2018 to March 2020, Pandemic: April 2020 to March 2022, Post-TCV: April 2022 to April 2024, m, Months, y, Years.
