# Supplementary figures and images for: Impact of the COVID-19 pandemic and typhoid conjugate vaccine introduction on typhoid fever in Nepal
Source: PLoS Negl Trop Dis. 2026 Jan 21;20(1):e0013242. doi: 10.1371/journal.pntd.0013242 (PMC12851488; doi:10.1371/journal.pntd.0013242)

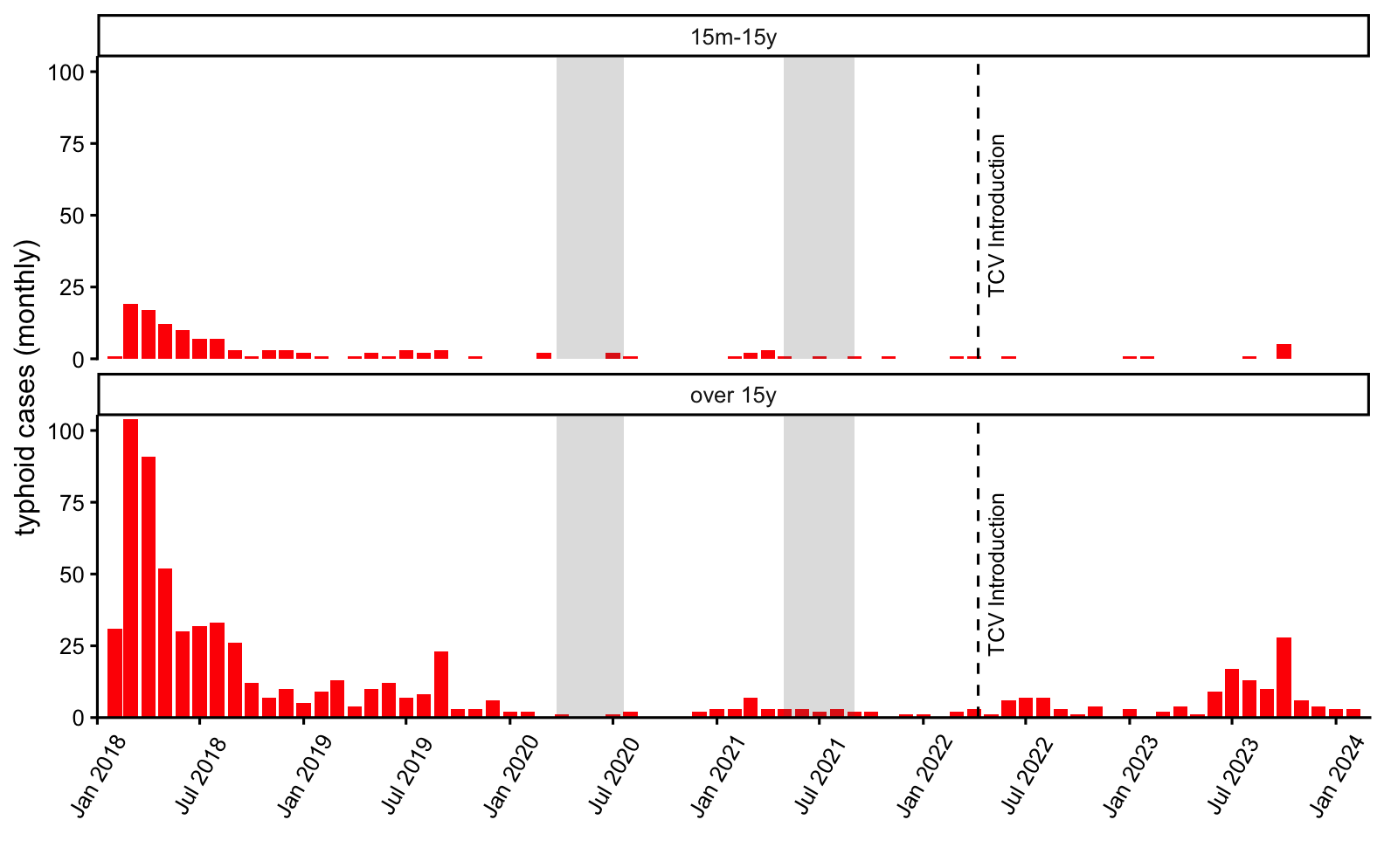

Supplement: S1 Fig — The top panel shows cases among children ages 15 months to 15 years, and the bottom panel shows cases in individuals over 15 years of age. The gray shaded areas reflect the time periods of the COVID-19 related lockdowns in Nepal. (PNG) [file pntd.0013242.s002.png]
