# Supplementary material for: Impact of the COVID-19 pandemic and typhoid conjugate vaccine introduction on typhoid fever in Nepal
Source: PLoS Negl Trop Dis. 2026 Jan 21;20(1):e0013242. doi: 10.1371/journal.pntd.0013242 (PMC12851488; doi:10.1371/journal.pntd.0013242)
Supplement: Attachment — Submitted filename: ResponsetoReviewers.pdf [file pntd.0013242.s005.pdf]

Manuscript PNTD-D-25-00973

Response to Reviewers

Dear Drs. Bowen, Blacksell, Kamwahi, and Brindley,

Thank you for giving us the opportunity to resubmit our manuscript titled "Impact of the COVID-19 pandemic and typhoid conjugate vaccine introduction on typhoid fever in Nepal." We appreciate the time and effort that you and the reviewers dedicated to providing insightful feedback on our manuscript and we are grateful for the opportunity to make these valuable improvements to our paper. In addition to updating the submission per journal requirements, we have incorporated the suggestions made by the reviewers, with all changes visible in the tracked version of the revised manuscript. Please see below, in **red**, for a point-by-point response to the reviewers' comments. All line and page numbers refer to the cleaned, revised manuscript file.

Reviewer #1

A well-written manuscript, few recommendations to improve it.

Response: We appreciate the reviewer's positive feedback and for their suggestions on how to improve the manuscript.

Methods

1) Further explanation on why those sites were chosen is required. This could have attracted a selection bias, in terms of better infrastructure, better adherence, …etc.

   Response 1: We thank the reviewer for the question on site selection. These sites were part of the Surveillance of Enteric Fever Asia Project study where the sites were selected on the basis of availability of blood culture facility and at least 20-30 cases of enteric fever positive per year. We included the four sites who had the retrospective record of demographic and blood cultural outcome. While our study focused on hospitals in and around the Kathmandu Valley, these sites represent a range of urban and peri-urban settings that together capture diversity in healthcare utilization and typhoid epidemiology within this region of Nepal. We have included more information on the site selection in the methods (lines 127-130):

   "Sites were selected on the basis of blood culture availability and at least 20-30 cases of enteric fever per year. For this sub-study, we included four sites that participated in SEAP since its inception in September 2016 and had retrospective records of demographic and blood culture outcomes."

   Additionally, we acknowledge that these sites do not fully capture the diversity of settings in Nepal. We have added the following to the discussion to address this limitation:

"Fourth, although our study included both urban and peri-urban hospitals, the findings may not be fully generalizable to all regions of Nepal, particularly rural areas with different healthcare access and transmission dynamics. However, these sites reflect risk in and around the Kathmandu Valley and provide early evidence on typhoid trends following TCV introduction."

2) Could you comment on the process of presenting to healthcare facilities- like GP referral or self-presentation.

   Response 2: We thank the reviewer for the opportunity to clarify the process of presenting to healthcare facilities. In Nepal, patients almost all self-present to these facilities, as there are not formal GP referral services. We have added the following to the methods to clarify this (lines 137-139):

   "In Nepal, patients typically self-present to healthcare facilities, as formal general practitioner referral services are not available in this setting."

3) More information on the regression analysis is required. How did you handle the variables in the regression analyses? How did you choose the final model? How did you make sure that it is the best fitting model?

   Response 3: We thank the reviewer for this helpful suggestion to clarify the regression analyses. There were limited individual data available for this analysis (month of collection, study site, age, etc). Confounders were selected based on data availability and a priori relevance. When we compared the AIC of the model with and without a fixed effect for month, the model with the fixed effect indicated best fit.

   We have expanded the methods section to address our modeling approach and variable selection (lines 184-188):

   "Models included fixed effects for months of blood culture collection, study site, and study period, all treated as categorical variables, with the former selected based on plausible confounding. Model selection was confirmed using Akaike Information Criterion (AIC), with the inclusion of month as a fixed effect improving model fit."

4) More information on how you safeguarded the confidentiality of the participants is good to be added to the ethical statement. Things like deidentification of data…etc

   Response 4: We agree with the reviewer on clarifying confidentiality safeguards:

   Lines 198-199: "Confidentiality of participant data was ensured by de-identifying all records and aggregating blood culture results prior to analysis."

Results

5) Most blood cultures were obviously pre-COVID. Could this have influenced your data?

   Response 5: We agree with the reviewer that most blood cultures were obtained before the COVID-19 pandemic. We recognize that this could influence overall counts and potentially confound comparisons across time, which motivated our use of culture

positivity rather than case counts We considered including overall testing volume (total blood cultures collected) in the regression models; however, this variable was highly collinear with study period, and its inclusion did not materially change the model fit or estimates. We have now clarified this in the manuscript in the Methods section:

Lines 184-188: “Models included fixed effects for months of blood culture collection, study site, and study period, all treated as categorical variables, with the former selected based on plausible confounding. Model selection was confirmed using Akaike Information Criterion (AIC), with the inclusion of month as a fixed effect improving model fit.”

6) You need to highlight that some values have wide CI and not significant, putting uncertainties on the value of the results. Example: “After TCV introduction, compared to the pandemic period, the odds of culture 252 positivity were 71% lower (aOR 0.29, 95% CI 0.05-1.35) in those ages 15 months to 15 years”

Response 6: We agree with the reviewer on providing uncertainty on estimates in the text and clarifying those that were not significant. We have implemented these changes in the last paragraph of the results:

“In sensitivity analysis, when restricting the analysis to blood cultures containing any pathogen, the results were similar. The odds of blood culture positivity for S. Typhi were 51% lower **but not statistically significant** (aOR 0.49, 95% CI 0.18-1.28) in those ages 15 months to 15 years and 86% lower (aOR 0.14, 95% CI 0.09-0.20) in those 16 and older during the pandemic compared to the pre-pandemic period. After TCV introduction, compared to the pandemic period, the odds of culture positivity were 71% lower **but not statistically significant** (aOR 0.29, 95% CI 0.05-1.36) in those ages 15 months to 15 years and 100% higher (aOR 2.00, 95% CI 1.33-3.06) in those 16 years and older (**Table 2**)”

Conclusion

7) I have concerns about the generalisability of the findings and particularly I wouldn’t draw a broader conclusion about the impact of the TCV role out, but rather these findings reflect these areas and only examined population-

Response 7: We thank the reviewer for their comments on the generalizability of our findings. While our study focused on hospitals in and around the Kathmandu Valley, these sites represent a range of urban and peri-urban settings that together capture diversity in healthcare utilization and typhoid epidemiology within this region of Nepal. We agree that our findings may not be generalizable to rural areas or settings with different transmission dynamics. We have added this as a limitation to the Discussion to clarify that the results reflect the experience of these surveillance sites and may be most applicable to similar urban and peri-urban contexts in Nepal:

Lines 336-340: Fourth, although our study included both urban and peri-urban hospitals, the findings may not be fully generalizable to all regions of Nepal, particularly rural areas with different healthcare access and transmission dynamics. However, these sites reflect

risk in and around the Kathmandu Valley and provide early evidence on typhoid trends following TCV introduction.

Reviewer #2

In "Impact of the COVID-19 pandemic and typhoid conjugate vaccine introduction on typhoid fever in Nepal," the authors investigated the impact of TCV introduction after the COVID pandemic in Nepal. This is a very timely study with interesting implications, and can fill some much-needed gaps about routine use of typhoid conjugate vaccines. That being said, some of the implications stated in the discussion are too strong given the limitations of the methods chosen and challenges in evaluating post-pandemic data. Based on these methods and data, it is not possible to say that the decline in typhoid is purely because of TCVs. I would suggest either softening some of these conclusions, or doing some additional or alternative analyses to be able to say this. I would also recommend doing a literature search on this topic before writing the manuscript in the future, as some of the citations could be updated.

Response: We appreciate the reviewer's recognition of the significance of this study and for their suggestions on how to improve the manuscript.

Introduction

- Consider citing
  - Antillon M, Warren J, Crawford F, Weinberger D, Kurum E, Pitzer V. The burden of typhoid fever in low- and middle-income countries: a meta-regression approach. PLoS Negl Trop Dis. 2017. https://doi.org/10.1371/journal.pntd.0005376.
  - GBD 2017 Risk Factor Collaborators. Global, regional, and national comparative risk assessment of 84 behavioural, environmental and occupational, and metabolic risks or clusters of risks for 195 countries and territories, 1990–2017: a systematic analysis for the Global Burden of Disease Study 2017. Lancet (London, England). 2018;392(10159):1923–94.
  - Mogasale V, Maskery B, Ochiai RL, et al. Burden of typhoid fever in low-income and middle-income countries: a systematic, literature-based update with risk-factor adjustment—the lancet global health. Lancet. 2014;2(10):e570-e580. 10.1016/S2214-109X(14)70301-8

  for annual burden estimate citations instead of the current citation for a single year (2021).

  Response 1: We thank the reviewer for providing these citations for annual burden estimates. We have updated the introduction and the references accordingly (lines 90-92):

  "Typhoid is a life-threatening systemic infection caused by Salmonella enterica serovar Typhi (S. Typhi), which causes an estimated 11-21 million illnesses and 135,000-200,000 deaths annually in low- and middle-income countries[1-3]."

- Please also consider citing:

- o Mila Shakya, Merryn Voysey, Katherine Theiss-Nyland, Rachel Colin-Jones, Dikshya Pant, Anup Adhikari, Susan Tonks, Yama F Mujadidi, Peter O'Reilly, Olga Mazur, Sarah Kelly, Xinxue Liu, Archana Maharjan, Ashata Dahal, Naheeda Haque, Anisha Pradhan, Suchita Shrestha, Manij Joshi, Nicola Smith, Jennifer Hill, Jenny Clarke, Lisa Stockdale, Elizabeth Jones, Timothy Lubinda, Binod Bajracharya, Sabina Dongol, Abhilasha Karkey, Stephen Baker, Gordan Dougan, Virginia E Pitzer, Kathleen M Neuzil, Shrijana Shrestha, Buddha Basnyat, Andrew J Pollard. Efficacy of typhoid conjugate vaccine in Nepal: final results of a phase 3, randomised, controlled trial. The Lancet Global Health. Volume 9, Issue 11. 2021. Pages e1561-e1568. https://doi.org/10.1016/S2214-109X(21)00346-6.

for efficacy of Phase 3 trial of TCVs in Nepal. There are also several others, but please at least include this one.

Response 2: We thank the reviewer for the updated Shakya et al. citation. We have included this in our updated references and incorporated the in-text citation throughout.

Methods

- How were the 4 sites chosen for this study? How representative are they of the general population? Three of the sites appear to be very close together and may have overlapping catchment areas, while one is several [districts?] away and may have a very different population.

  Response 3: We thank the reviewer for the question on site selection. These sites were part of the Surveillance of Enteric Fever Asia Project study where the sites were selected on the basis of availability of blood culture facilities and at least 20-30 cases of enteric fever positive per year. We included the four sites who had the retrospective record of demographic and blood cultural outcome. While our study focused on hospitals in and around the Kathmandu Valley, these sites represent a range of urban and peri-urban settings that together capture diversity in healthcare utilization and typhoid epidemiology within this region of Nepal.

  However, we acknowledge that these sites do not fully capture the diversity of settings in Nepal. We have added the following to the discussion to address this limitation:

  "Fourth, although our study included both urban and peri-urban hospitals, the findings may not be fully generalizable to all regions of Nepal, particularly rural areas with different healthcare access and transmission dynamics. However, these sites reflect risk in and around the Kathmandu Valley and provide early evidence on typhoid trends following TCV introduction."

- Please mention which TCV was used in Nepal in the Methods.
  Response 4: We agree with the reviewer that the TCV should be specified in the methods, and have updated lines 160-161 to do so:

  "Nepal introduced TyphiBEV® (Vi-CRM197), a typhoid conjugate vaccine, into its national immunization program on April 13, 2022."

- Can the authors include the exact date of TCV introduction in this area in the Methods? Also, why is April 2022 part of the pandemic ("prior to TCV introduction" Lines 170-171) period when it was when the vaccine was introduced?

  Response 5: We agree with the reviewer that the exact date of TCV introduction should be specified methods, and have updated lines 160-161 to do so:

  "Nepal introduced TyphiBEV® (Vi-CRM197), a typhoid conjugate vaccine, into its national immunization program on April 13, 2022."

  We also agree that April 2020-April 2022 being labeled as the pandemic is confusing as the latter date is excluded in that period, so we have updated the time period to "April 2020-March 2022" throughout the manuscript.

- The authors could consider doing a sensitivity analysis with a pre-pandemic period excluding 2018.

  Response 6: We thank the reviewer for this important suggestion. We have run a sensitivity analysis excluding 2018 in the pre-pandemic period (N = 50,037).

  This analysis revealed that as explained, 2018 was an outlier year with high typhoid incidence. The third limitation has been updated in the text (lines 333-336) to reflect this:

  "Third, S. Typhi cases were unusually high in 2018 compared with prior years, such that the decline during and after 2020 was accentuated; nevertheless, compared with data from earlier years (Figure 1), S. Typhi cases were still markedly lower during the COVID-19 period"

**Denominator: All blood cultures**			
		**aOR**	**95% CI**
15m-15y	Pandemic v. Pre-pandemic w/o 2018	**2.22**	**1.07-4.55**
	Post-TCV v. Pandemic	0.17	0.07-0.39
≥16y	Pandemic v. Pre-pandemic w/o 2018	0.81	**0.56-1.15**
	Post-TCV v. Pandemic	1.32	**0.94-1.90**
**Denominator: Blood cultures containing any pathogen**			
		**aOR**	**95% CI**
15m-15y	Pandemic v. Pre-pandemic w/o 2018	**2.44**	**0.70-8.59**
	Post-TCV v. Pandemic	0.09	0.00-0.63
≥16y	Pandemic v. Pre-pandemic w/o 2018	0.45	0.29-0.70
	Post-TCV v. Pandemic	1.72	1.14-2.64

- How long was the catchup campaign? And how long did it take to implement? It may not make sense to use the beginning of some of the post-pandemic vaccination period.

Response 7: The catchup campaign was implemented for one month (April 2022), followed by introduction of the routine vaccination, thus we felt it was most appropriate to include April as the vaccine period.

- Did everyone consent to enrollment? (what was the participation rate)

Response 8: We used retrospective, de-identified data on age and outcome of the blood culture from laboratory records, so no informed consent was sought. There was less than 1 percent missing the required information. Local site and national IRB approval were received for the study. This is clarified in the "Ethical considerations" section.

- The authors could consider including study site as a random effect and then looking at the within and between study site effects (in the logistic regression).

Response 9: We thank the reviewer for this suggestion. Below, we have refitted our models using mixed effects logistic regression with study site as a random effect rather than a fixed effect, to account for clustering of observations within sites. The results were similar to our original fixed effects models and did not materially change our conclusions.

**Denominator: All blood cultures**			
		**aOR**	**95% CI**
15m-15y	Pandemic v. Pre-pandemic	0.53	0.30-0.92
	Post-TCV v. Pandemic	0.25	0.11-0.56
≥16y	Pandemic v. Pre-pandemic	0.23	0.17-0.31
	Post-TCV v. Pandemic	1.60	1.13-2.26
**Denominator: Blood cultures containing any pathogen**			
		**aOR**	**95% CI**
15m-15y	Pandemic v. Pre-pandemic	0.50	0.19-1.29
	Post-TCV v. Pandemic	0.35	0.08-1.62
≥16y	Pandemic v. Pre-pandemic	0.14	0.09-0.20
	Post-TCV v. Pandemic	2.00	1.32-3.03

Results

- Fig 2: It might make sense to convert the COVID-19 cases to monthly instead of daily, to match the other plots. Can the authors add what the different colors represent in the caption? Also, it would be really helpful to see all of the time series (especially the last one, typhoid cases) by study site. Can the authors also show a plot with a smaller y-axis (i.e., excluding 2018) so we can see the lower case count trends more clearly? As it is, it is very difficult to see the case numbers and patterns after 2020.

Response 10: We thank the reviewer for their comments on Figure 2. We have converted the COVID-19 cases to monthly, and added the different colors to the caption:

Fig 2. Trends of monthly COVID-19 cases, blood culture testing, *Salmonella* Typhi cases, and other bacteremia cases (2016-2024).

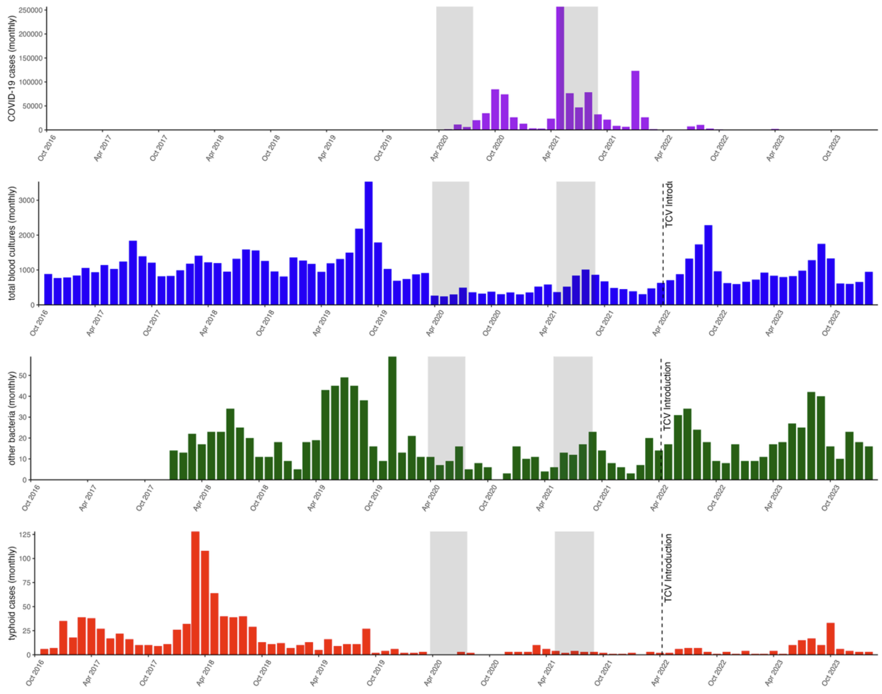

The gray shaded areas reflect the time periods of the COVID-19 related lockdowns in Nepal. Purple: total COVID-19 cases, blue: total blood cultures collected, green: total other bacteremia cases, red: total other typhoid cases.

Additionally, we have attached a plot without 2018 for the typhoid case counts, and the cases by site. For conciseness and readability, we have not included all time series by site in the main figures.

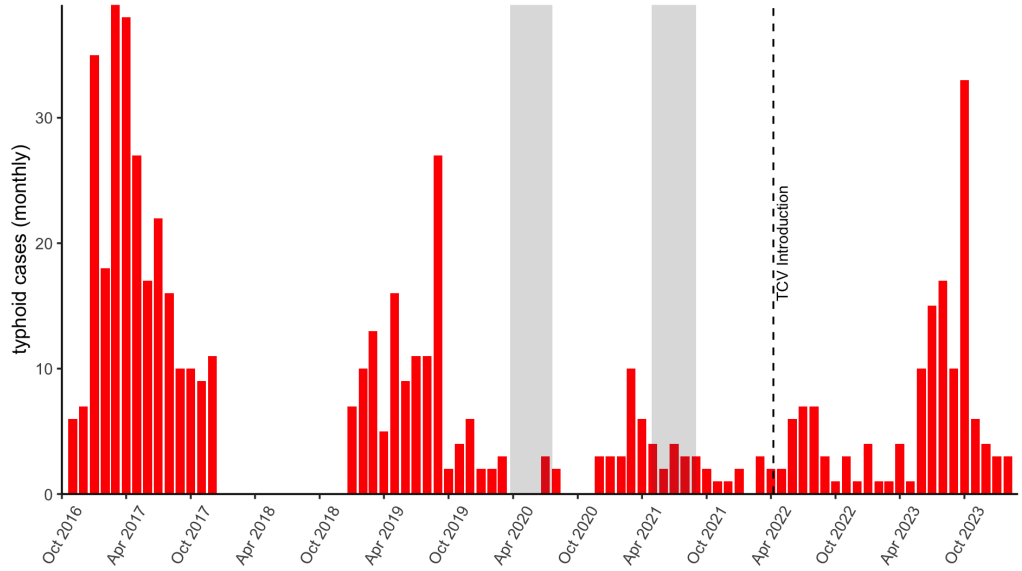

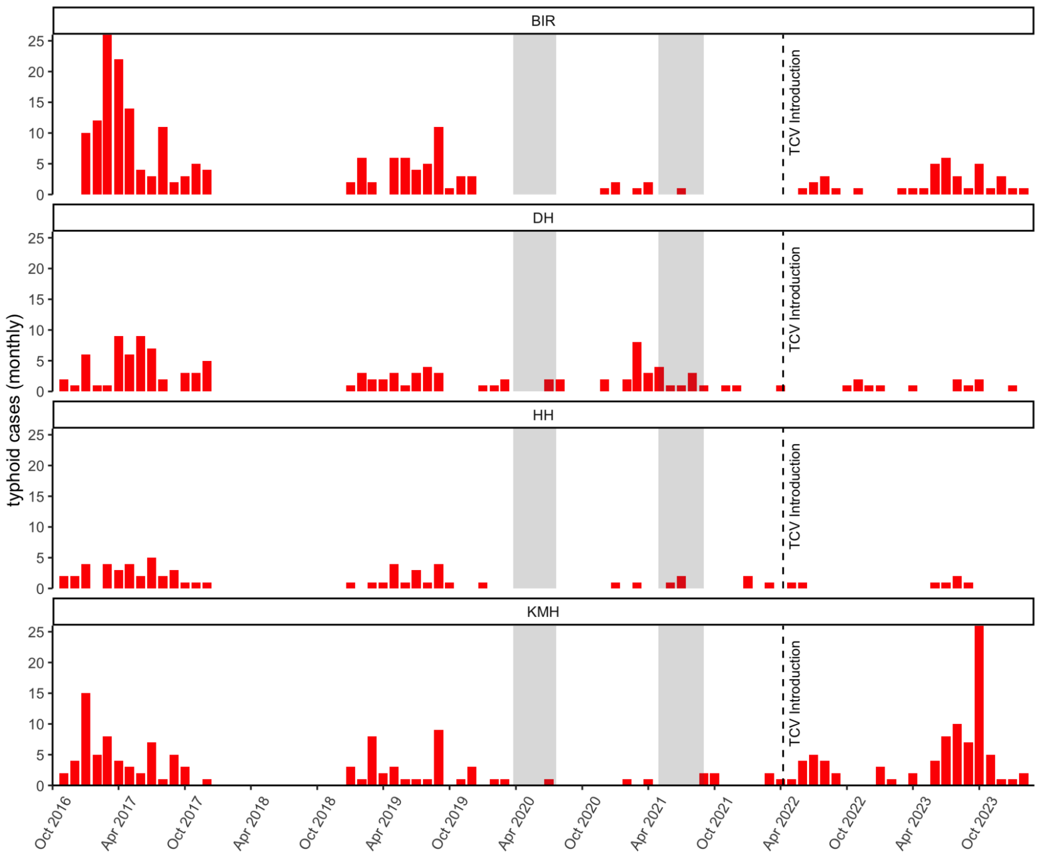

- It almost looks like cases increased after TCV introduction (by looking at Fig 2). It might be more helpful to also look at impact by year (even within each pre/during/post pandemic period) to see if it is stable within each period. Could do something like a synthetic control using other pathogens if they had a similar pre-intervention trends.

  Response 11: We explored an interrupted time series analysis to assess whether temporal trends of cases differed by period. We modeled monthly trends using exclusive period indicators and period-specific slopes, adjusting for site and age group.

  For ages 15 months to 15 years, ITS analysis indicated that before COVID, the odds of Typhi detection were declining (OR per month = 0.90, 95% CI 0.88-0.93). At the onset of the COVID-19 pandemic, there was an immediate drop in odds relative to the pre-COVID period (OR = 0.08, 95% CI 0.02-0.21), with an approximately flat slope during the COVID pre-vaccination period (OR per month = 0.99, 95% CI 0.91-1.07). Following TCV introduction, there was a further immediate decrease in odds (OR = 0.02, 95% CI 0.01-0.06), with an insignificant decline in the post-TCV period (OR per month = 0.96, 95% CI 0.88-1.04).

  For ages 16 and older, ITS analysis indicated that before COVID, the odds of Typhi detection were declining (OR per month = 0.89, 95% CI 0.88-0.90). At the onset of the COVID-19 pandemic, there was an immediate drop in odds relative to the pre-COVID

period (OR = 0.05, 95% CI 0.02-0.10), with an approximately flat slope during the COVID pre-vaccination period (OR per month = 1.00, 95% CI 0.95-1.05). Following TCV introduction, there was a further immediate decrease in odds (OR = 0.04, 95% CI 0.03-0.06), with a slight upward trend in the post-TCV period (OR per month = 1.03, 95% CI 1.00-1.05), consistent with the apparent rebound observed in Fig. 2.

These ITS findings align with our main results: while both age groups experienced sharp declines in *S. Typhi* positivity during the COVID-19 pandemic, only the vaccine-ineligible older group showed a rebound after 2022, whereas cases continued to decline in vaccine-eligible children following TCV introduction.

- It would be helpful to see the results for other pathogens (like Table 2 results) over the same time periods and in the same groups for comparison.

Response 12: We thank the reviewer for this helpful suggestion. We have attached the results for the other pathogens (other bacteria including Paratyphi) below:

**Denominator: All blood cultures**			
		**aOR**	**95% CI**
15m-15y	Pandemic v. Pre-pandemic	0.89	0.49-1.53
	Post-TCV v. Pandemic	0.39	0.18-0.81
≥16y	Pandemic v. Pre-pandemic	1.20	1.02-1.69
	Post-TCV v. Pandemic	0.98	0.84-1.16

We acknowledge that these results show a decrease in positivity for other pathogens among vaccine-eligible children (15m-15y) following TCV introduction. This concurrent decline suggests that factors other than TCV introduction could have influenced blood culture positivity in this period. We have added a statement acknowledging this as a major limitation of our analysis, as we are unable to fully explain the concurrent decrease in other bacterial pathogens.

Lines 350-355: Although the reduction in *S. Typhi* positivity among vaccine-eligible individuals following TCV introduction is consistent with vaccine impact, we also observed a concurrent decline in positivity for other bacteria in this age group. This pattern indicates that some of the observed reduction may reflect broader changes beyond TCV rollout, and we are unable to fully explain these findings. We therefore interpret the post-TCV decline with caution and acknowledge this as a major limitation of our analysis.

- What were the estimates for the adjustment factors in the logistic regression? Were there any substantial differences between sites (and other variables)? What were those impacts and what did they look like? Again, the authors could consider a random effect for study site or something similar.

Response 13: We thank their reviewer for their questions on the regression analysis. Per our analysis in Response 9, there was no substantial difference when considering a random effect for study site:

Dhulikhel Hospital (DH): aOR 0.23 (95% CI 0.13-0.45)
Helping Hands (HH): aOR 0.86 (95% CI 0.45-1.74)
Kathmandu Model Hospital (KMH): aOR 1.01 (95% CI 0.53-2.04)

Discussion

- Some of the implications stated in the discussion are too strong, given the limitations of the methods chosen and challenges in evaluating post-pandemic data. Since the pandemic and post-pandemic cases remain low, the authors cannot state that the lower cases post-pandemic were only because of vaccination. This is a limitation of the data, but it would be possible to use other pathogens (or other non-intervention sites) over the same time period. This additional analysis is not mandatory, but these limitations need to be acknowledged further and the conclusions need to be softened if no additional analyses are included.

  Response 14: We thank the reviewer for their suggestion for the implications of the study. We agree that our data have major limitations, and we have revised the discussion accordingly (lines 350-355):

  Although the reduction in *S. Typhi* positivity among vaccine-eligible individuals following TCV introduction is consistent with vaccine impact, we also observed a concurrent decline in positivity for other bacteria in this age group. This pattern indicates that some of the observed reduction may reflect broader changes beyond TCV rollout, and we are unable to fully explain these findings. We therefore interpret the post-TCV decline with caution and acknowledge this as a major limitation of our analysis.

- Consider citing these studies in the limitations where the authors mention antibiotic use and its influence on blood culture sensitivity:
  - Antillon M, Saad NJ, Baker S, Pollard AJ, Pitzer VE. The relationship between blood sample volume and diagnostic sensitivity of blood culture for typhoid and paratyphoid fever: a systematic review and meta-analysis. J Infect Dis. 2018;218(suppl_4):S255-S267. 10.1093/infdis/jiy471
  - Phillips MT, Meiring JE, Voysey M, Warren JL, Baker S, Basnyat B, Clemens JD, Dolecek C, Dunstan SJ, Dougan G, Gordon MA, Thindwa D, Heyderman RS, Holt KE, Qadri F, Pollard AJ, Pitzer VE; STRATAA Study Group. A Bayesian approach for estimating typhoid fever incidence from large-scale facility-based passive surveillance data. Stat Med. 2021 Nov 20;40(26):5853-5870. doi: 10.1002/sim.9159. Epub 2021 Aug 24. PMID: 34428309; PMCID: PMC9291985.

  These indicate that antibiotic use can greatly impact blood culture sensitivity and reporting. In Figure S2 of the second citation, not that Nepal has some of the highest self-reported antibiotic use.

Response 15: We thank the reviewer for providing these citations regarding antibiotic use and blood culture. We have updated the discussion's references accordingly.

- Consider exploring or commenting on how reporting might have changed through the different periods, and how it might vary by study sites. This may impact the findings.

  Response 16: We thank the reviewer for their comments on changes in reporting and testing capacity throughout the periods. During the study period, the reporting mechanism was not changed, but we considered including overall testing volume (total blood cultures collected) in the regression models; however, this variable was highly collinear with study period, and its inclusion did not materially change the model fit or estimates.

  We have now clarified this in the manuscript in the Methods section:
  Lines 184-188: "Models included fixed effects for months of blood culture collection, study site, and study period, all treated as categorical variables, with the former selected based on plausible confounding. Model selection was confirmed using Akaike Information Criterion (AIC), with the inclusion of month as a fixed effect improving model fit."

- Similar to the previous comment, are there differences between the reporting (or underreporting) of prospective and retrospective sites/time periods?

  Response 17: We thank the reviewer for pointing this out, and we have added the following comment to the discussion section to acknowledge the fact that the prospective enrollment at DH and KMH hospitals could have an impact (lines 340-345):

  "Our analysis included sites that had both prospective enrollment of febrile patients and retrospective enrollment based on blood cultures routinely performed by the laboratory. Prospective enrollment increased the number of blood cultures performed at a site. We used fixed effects for each site to account for differences between sites and analyzed culture positivity rather than case counts, but differences over time might have still impacted the results."

- How do these results compare to previous modeling studies about vaccine implementation in similar settings (like this: Joke Bilcke, Marina Antillón, Zoë Pieters, Elise Kuylen, Linda Abboud, Kathleen M Neuzil, Andrew J Pollard, A David Paltiel, Virginia E Pitzer. Cost-effectiveness of routine and campaign use of typhoid Vi-conjugate vaccine in Gavi-eligible countries: a modelling study. The Lancet Infectious Diseases. Volume 19, Issue 7, 2019, Pages 728-739. https://doi.org/10.1016/S1473-3099(18)30804-1)?

  Response 18: Our study evaluates real-world trends of typhoid cases upon implementation of a routine vaccination program with a catch-up campaign. In the Blicke et al. modeling study, the predicted cases averted for Nepal upon implementation of routine vaccination with a catch-up campaign up to 15 years of age was 438,000 (155,000-828,000), amounting to nearly 50-70% of the 745,000 (320,000-1,183,000) predicted cases in the absence of vaccination. Among the TCV-eligible children in our

study (15 months to 15 years), we observed a 75% decrease in the odds of *S.* Typhi positivity post-TCV introduction, compared to positivity prior to rollout during the pandemic. Both studies reflect substantial impact in the vaccinated age group upon implementation of routine vaccination with a catch-up campaign.

- Can the authors add some more sentences about the broader public health implications of this study? For example, they mention the need for additional control measures like improved water and sanitation infrastructure in the abstract and hint at it in the introduction, but it isn't in the discussion. Could cite this paper: Phillips MT, Owers KA, Grenfell BT, Pitzer VE (2021). Changes in historical typhoid transmission across 16 U.S. cities, 1889–1931: Quantifying the impact of investments in water and sewer infrastructures. PLOS Neglected Tropical Diseases 15(4): e0009347. https://doi.org/10.1371/journal.pntd.0009347 .

  Response 19: We thank the reviewer for their comment on expanding the implications of this study. We have incorporated the citation in a sentence regarding control measures in the conclusion (lines 362-364):

  "Comprehensive control requires sustainable investments in water and sanitation infrastructure[34], alongside consideration of expanded age eligibility for typhoid vaccination."

- In the limitations, the authors could mention something about multidrug resistant strains of typhoid (optional).

  Response 20: Thank you for this question. In our surveillance in Nepal, fewer than 2% of strains are multidrug-resistant in recent years, and we believe this is not likely to substantially impact transmission dynamics or the study results.

- Can the authors state something about the long-term effects of the vaccine that aren't observed yet in this study (because it only spans ~2 years post vaccination)?

  Response 21: We agree with the reviewer's suggestion, and we have added this statement in lines 323-325:

  "Additionally, our study period ends approximately two years following TCV introduction, limiting our ability to assess longer-term vaccine effectiveness and durability of protection."

Minor

- Please spell out acronyms (like aOR and CI) before using them the first time.
  Response 23. We have now spelled out aOR and CI before using them the first time.

- Please be consistent with commas and decimal points in numbers (sometimes there are commas in the larger numbers and sometimes there aren't, and numbers after the decimals vary from 0-2 throughout the text and tables).

Response 24. We have revised the manuscript to ensure consistency in how numbers are presented.

- Please provide more detailed table and figure titles and captions. The titles should be able to stand alone—please give study setting and dates.

  Response 25: We have revised the table and figure titles to include study setting and dates.
