# Supplementary material for: Impact of the COVID-19 pandemic and typhoid conjugate vaccine introduction on typhoid fever in Nepal
Source: PLoS Negl Trop Dis. 2026 Jan 21;20(1):e0013242. doi: 10.1371/journal.pntd.0013242 (PMC12851488; doi:10.1371/journal.pntd.0013242)
Supplement: Attachment — Submitted filename: Review_ Impact of COVID-19 and TCVs Nepal.docx [file pntd.0013242.s003.docx]

In “Impact of the COVID-19 pandemic and typhoid conjugate vaccine introduction on typhoid fever in Nepal,” the authors investigated the impact of TCV introduction after the COVID pandemic in Nepal. This is a very timely study with interesting implications, and can fill some much-needed gaps about routine use of typhoid conjugate vaccines. That being said, some of the implications stated in the discussion are too strong given the limitations of the methods chosen and challenges in evaluating post-pandemic data. Based on these methods and data, it is not possible to say that the decline in typhoid is purely because of TCVs. I would suggest either softening some of these conclusions, or doing some additional or alternative analyses to be able to say this. I would also recommend doing a literature search on this topic before writing the manuscript in the future, as some of the citations could be updated.

**Introduction**

- Consider citing
  - Antillon M, Warren J, Crawford F, Weinberger D, Kurum E, Pitzer V. The burden of typhoid fever in low- and middle-income countries: a meta-regression approach. PLoS Negl Trop Dis. 2017. <https://doi.org/10.1371/journal.pntd.0005376>.
  - GBD 2017 Risk Factor Collaborators. Global, regional, and national comparative risk assessment of 84 behavioural, environmental and occupational, and metabolic risks or clusters of risks for 195 countries and territories, 1990–2017: a systematic analysis for the Global Burden of Disease Study 2017. Lancet (London, England). 2018;392(10159):1923–94.
  - Mogasale V, Maskery B, Ochiai RL, et al. Burden of typhoid fever in low‐income and middle‐income countries: a systematic, literature‐based update with risk‐factor adjustment—the lancet global health. Lancet. 2014;2(10):e570‐e580. 10.1016/S2214-109X(14)70301-8

for annual burden estimate citations instead of the current citation for a single year (2021).

- Please also consider citing:
  - Mila Shakya, Merryn Voysey, Katherine Theiss-Nyland, Rachel Colin-Jones, Dikshya Pant, Anup Adhikari, Susan Tonks, Yama F Mujadidi, Peter O’Reilly, Olga Mazur, Sarah Kelly, Xinxue Liu, Archana Maharjan, Ashata Dahal, Naheeda Haque, Anisha Pradhan, Suchita Shrestha, Manij Joshi, Nicola Smith, Jennifer Hill, Jenny Clarke, Lisa Stockdale, Elizabeth Jones, Timothy Lubinda, Binod Bajracharya, Sabina Dongol, Abhilasha Karkey, Stephen Baker, Gordan Dougan, Virginia E Pitzer, Kathleen M Neuzil, Shrijana Shrestha, Buddha Basnyat, Andrew J Pollard. Efficacy of typhoid conjugate vaccine in Nepal: final results of a phase 3, randomised, controlled trial. The Lancet Global Health. Volume 9, Issue 11. 2021. Pages e1561-e1568. https://doi.org/10.1016/S2214-109X(21)00346-6.

for efficacy of Phase 3 trial of TCVs in Nepal. There are also several others, but please at least include this one.

**Methods**

- How were the 4 sites chosen for this study? How representative are they of the general population? Three of the sites appear to be very close together and may have overlapping catchment areas, while one is several [districts?] away and may have a very different population.
- Please mention which TCV was used in Nepal in the Methods.
- Can the authors include the exact date of TCV introduction in this area in the Methods? Also, why is April 2022 part of the pandemic (“prior to TCV introduction” Lines 170-171) period when it was when the vaccine was introduced?
- The authors could consider doing a sensitivity analysis with pre-pandemic period excluding 2018.
- How long was the catchup campaign? And how long did it take to implement? It may not make sense to use the beginning of some of the post-pandemic vaccination period.
- Did everyone consent to enrolment? (what was the participation rate)
- The authors could consider including study site as a random effect and then looking at the within and between study site effects (in the logistic regression).

**Results**

- Fig 2: It might make sense to convert the COVID-19 cases to monthly instead of daily, to match the other plots. Can the authors add what the different colors represent in the caption? Also, it would be really helpful to see all of the time series (especially the last one, typhoid cases) by study site. Can the authors also show a plot with a smaller y-axis (i.e., excluding 2018) so we can see the lower case count trends more clearly? As it is, it is very difficult to see the case numbers and patterns after 2020.
- It almost looks like cases increased after TCV introduction (by looking at Fig 2). It might be more helpful to also look at impact by year (even within each pre/during/post pandemic period) to see if it is stable within each period. Could do something like a synthetic control using other pathogens if they had a similar pre-intervention trends.
- It would be helpful to see the results for other pathogens (like Table 2 results) over the same time periods and in the same groups for comparison.
- What were the estimates for the adjustment factors in the logistic regression? Were there any substantial differences between sites (and other variables)? What were those impacts and what did they look like? Again, the authors could consider a random effect for study site or something similar.

**Discussion**

- Some of the implications stated in the discussion are too strong, given the limitations of the methods chosen and challenges in evaluating post-pandemic data. Since the pandemic and post-pandemic cases remain low, the authors cannot state that the lower cases post-pandemic were only because of vaccination. This is a limitation of the data, but it would be possible to use other pathogens (or other non-intervention sites) over the same time period. This additional analysis is not mandatory, but these limitations need to be acknowledged further and the conclusions need to be softened if no additional analyses are included.
- Consider citing these studies in the limitations where the authors mention antibiotic use and its influence on blood culture sensitivity:
  - Antillon M, Saad NJ, Baker S, Pollard AJ, Pitzer VE. The relationship between blood sample volume and diagnostic sensitivity of blood culture for typhoid and paratyphoid fever: a systematic review and meta‐analysis. J Infect Dis. 2018;218(suppl_4):S255‐S267. 10.1093/infdis/jiy471
  - Phillips MT, Meiring JE, Voysey M, Warren JL, Baker S, Basnyat B, Clemens JD, Dolecek C, Dunstan SJ, Dougan G, Gordon MA, Thindwa D, Heyderman RS, Holt KE, Qadri F, Pollard AJ, Pitzer VE; STRATAA Study Group. A Bayesian approach for estimating typhoid fever incidence from large-scale facility-based passive surveillance data. Stat Med. 2021 Nov 20;40(26):5853-5870. doi: 10.1002/sim.9159. Epub 2021 Aug 24. PMID: 34428309; PMCID: PMC9291985.

These indicate that antibiotic use can greatly impact blood culture sensitivity and reporting. In Figure S2 of the second citation, not that Nepal has some of the highest self-reported antibiotic use.

- Consider exploring or commenting on how reporting might have changed through the different periods, and how it might vary by study sites. This may impact the findings.
- Similar to the previous comment, are there differences between the reporting (or underreporting) of prospective and retrospective sites/time periods?
- How do these results compare to previous modeling studies about vaccine implementation in similar settings (like this: Joke Bilcke, Marina Antillón, Zoë Pieters, Elise Kuylen, Linda Abboud, Kathleen M Neuzil, Andrew J Pollard, A David Paltiel, Virginia E Pitzer. Cost-effectiveness of routine and campaign use of typhoid Vi-conjugate vaccine in Gavi-eligible countries: a modelling study. The Lancet Infectious Diseases. Volume 19, Issue 7, 2019, Pages 728-739. <https://doi.org/10.1016/S1473-3099(18)30804-1>)?
- Can the authors add some more sentences about the broader public health implications of this study? For example, they mention the need for additional control measures like improved water and sanitation infrastructure in the abstract and hint at it in the introduction, but it isn’t in the discussion. Could cite this paper: Phillips MT, Owers KA, Grenfell BT, Pitzer VE (2021). Changes in historical typhoid transmission across 16 U.S. cities, 1889–1931: Quantifying the impact of investments in water and sewer infrastructures. PLOS Neglected Tropical Diseases 15(4): e0009347. <https://doi.org/10.1371/journal.pntd.0009347> .
- In the limitations, the authors could mention something about multidrug resistant strains of typhoid (optional).
- Can the authors state something about the long-term effects of the vaccine that aren’t observed yet in this study (because it only spans ~2 years post vaccination)?

**Minor**

- Please spell out acronyms (like aOR and CI) before using them the first time.
- Please be consistent with commas and decimal points in numbers (sometimes there are commas in the larger numbers and sometimes there aren’t, and numbers after the decimals vary from 0-2 throughout the text and tables).
- Please provide more detailed table and figure titles and captions. The titles should be able to stand alone—please give study setting and dates.
